# Adaptations to comprehensive abortion care during the COVID-19 pandemic: case studies of provision in Bolivia, Mali, Nepal, and the occupied Palestinian territory

**DOI:** 10.1080/26410397.2023.2249694

**Published:** 2023-09-25

**Authors:** Annik Mahalia Sorhaindo, Sarah Castle, Lola Flomen, Eva Lathrop, Shirine Mohagheghpour, Rasha Dabash, Francelle Kwankam Toedtli, Rebecca Wilkins, Laurence Läser, Patricia Titulaer, Ernest Nyamato, Mary Lea Dakouo, Ammal Awadallah, Raman Shrestha, Malena Morales, Ulrika Rehnström Loi

**Affiliations:** a Technical Consultant, UNDP-UNFPA-UNICEF-WHO-World Bank Special Programme of Research, Development and Research Training in Human Reproduction (HRP), Department of Sexual and Reproductive Health and Research, World Health Organization, Geneva, Switzerland; b Technical Consultant, UNDP-UNFPA-UNICEF-WHO-World Bank Special Programme of Research, Development and Research Training in Human Reproduction (HRP), Department of Sexual and Reproductive Health and Research, World Health Organization, Geneva, Switzerland; c Mixed Health Systems Consultant, Population Services International, Washington DC, USA; d Global Medical Director, Population Services International, Washington DC, USA; e Senior Technical Advisor for Service Delivery, Population Services International, Washington DC, USA; f Senior Technical Consultant, Ipas, Chapel Hill, NC, USA; g Technical Specialist for SRHR and MNH, UNFPA, New York, USA; h Technical Lead, Abortion, International Planned Parenthood Federation, London, UK; i Technical Officer UNDP-UNFPA-UNICEF-WHO-World Bank Special Programme of Research, Development and Research Training in Human Reproduction (HRP), Department of Sexual and Reproductive Health and Research, World Health Organization, Geneva, Switzerland; j Technical Consultant, UNDP-UNFPA-UNICEF-WHO-World Bank Special Programme of Research, Development and Research Training in Human Reproduction (HRP), Department of Sexual and Reproductive Health and Research, World Health Organization, Geneva, Switzerland; k Global Associate Director, Quality of Care, Ipas, Nairobi County, Kenya; l Senior Technical Advisor, Population Services International, Bamako, Mali; m Executive Director, Palestine Family Planning and Protection Association (PFPPA), Jerusalem, Israel; n Global Evidence and Impact Advisor, Marie Stopes Nepal, Baluwatar, Kathmandu, Nepal; o Country Director Bolivia, Ipas LAC Region, La Paz, Bolivia; p Technical Officer, UNDP-UNFPA-UNICEF-WHO-World Bank Special Programme of Research, Development and Research Training in Human Reproduction (HRP), Department of Sexual and Reproductive Health and Research, World Health Organization, 20 Avenue Appia, 1211 Geneva, Switzerland.

**Keywords:** medical abortion, service adaptation, COVID-19, Nepal, Mali, Bolivia, occupied Palestinian territory, digital technologies

## Abstract

The COVID-19 pandemic impacted comprehensive abortion care provision. To maintain access to services while keeping individuals safe from infection, many organisations adapted their programmes. We conducted a programme evaluation to examine service adaptations implemented in Bolivia, Mali, Nepal, and the occupied Palestinian territory. Our programme evaluation used a case study approach to explore four programme adaptations through 14 group and individual interviews among 16 service providers, facility managers and representatives from supporting organisations. Data collection took place between October 2021 and January 2022. We identified adaptations to comprehensive abortion care services in relation to provision, health information systems and counselling, and referrals. Four overarching strategies emerged: (1) the use of digital technologies, (2) home and community outreach, (3) health worker optimisation, and (4) further consideration of groups in vulnerable situations. In Bolivia, the use of a messaging application increased access to confidential gender-based violence support and comprehensive abortion care. In Mali, the adoption of digital approaches created timely and complete data reporting and trained members of the community served as “interlocutors” between the communities and providers. In Nepal, an interim law expanded medical abortion provision to pharmacies, and home visits complemented facility-based services. In the occupied Palestinian territory, the use of a hotline and social media expanded access to quick and reliable information, counselling, referrals, and post-abortion care. Adaptations to comprehensive abortion care service delivery to mitigate disruptions to services during the COVID-19 pandemic may continue to benefit service quality of care, access to care, routine monitoring, as well as inclusivity and communication in the longer term.

## Résumé

La pandémie de COVID-19 a eu des conséquences sur la prestation de soins complets pour avortement. Afin de maintenir l’accès aux services tout en protégeant les individus contre l’infection, beaucoup d’organisations ont adapté leurs programmes. Nous avons mené une évaluation des programmes pour examiner les adaptations des services mises en œuvre en Bolivie, au Mali, au Népal et dans le territoire palestinien occupé. Notre évaluation a utilisé une approche d’étude de cas pour explorer quatre adaptations de programme par le biais de 14 entretiens individuels et de groupe auprès de 16 prestataires de services, directeurs d’établissement et représentants d’organisations de soutien. Nous avons identifié les adaptations aux services de soins complets pour avortement en rapport avec la prestation des services, les systèmes d’information sur la santé, le conseil et les aiguillages vers d’autres services. Quatre stratégies d’ensemble sont apparues: (1) l’utilisation de technologies numériques, (2) des services de proximité à domicile et dans la communauté, (3) l’optimisation des agents de santé et (4) une prise en compte plus approfondie des groupes vulnérables. En Bolivie, l’utilisation d’une application de messagerie instantanée a élargi l’accès à un soutien confidentiel en matière de violence sexiste et à des soins complets pour avortement. Au Mali, l’adoption d’approches numériques a créé une communication rapide et complète des données et les membres formés de la communauté ont servi d’« interlocuteurs » entre les communautés et les prestataires. Au Népal, une loi provisoire a étendu la prestation de l’avortement médicamenteux aux pharmacies et les visites à domicile ont complété les services assurés dans les centres. Dans le territoire palestinien occupé, l’utilisation d’un service d’assistance téléphonique et des médias sociaux a élargi l’accès à des informations rapides et dignes de foi, aux conseils, à l’aiguillage vers d’autres prestataires et aux soins après avortement. Les adaptations de la prestation de services de soins complets pour avortement afin d’atténuer les perturbations des services pendant la pandémie de COVID-19 peuvent continuer à bénéficier à la qualité des services, à l’accès aux soins, au suivi systématique, ainsi qu’à l’inclusivité et à la communication à long terme.

## Resumen

La pandemia de COVID-19 afectó la prestación de servicios de atención integral al aborto. Para mantener el acceso a los servicios y evitar que las personas se infectaran con el virus, muchas organizaciones adaptaron sus programas. Realizamos una evaluación del programa para examinar las adaptaciones de servicios aplicadas en Bolivia, Mali, Nepal y el territorio palestino ocupado. La evaluación del programa utilizó el enfoque de estudio de casos para explorar cuatro adaptaciones del programa por medio de 14 entrevistas en grupo e individuales con 16 prestadores de servicios, administradores de establecimientos de salud y representantes de organizaciones solidarias. Identificamos adaptaciones a los servicios de atención integral al aborto con relación a la prestación, sistemas de información sobre salud, consejería y referencias. Surgieron cuatro estrategias generales: (1) el uso de tecnologías digitales, (2) extensión domiciliaria y comunitaria, (3) optimización de trabajadores de salud, y (4) mayor consideración de grupos en situaciones vulnerables. En Bolivia, el uso de una aplicación de mensajes aumentó el acceso al apoyo confidencial en situaciones de violencia de género y a la atención integral al aborto. En Mali, la adopción de enfoques digitales facilitó la rendición de informes de datos oportunos y completes, e integrantes de la comunidad capacitados sirvieron como “interlocutores” entre sus comunidades y prestadores de servicios. En Nepal, una ley interina extendió la provisión de aborto con medicamentos a farmacias, y visitas domiciliarias suplementaron los servicios proporcionados en establecimientos de salud. En el territorio palestino ocupado, el uso de una línea de atención telefónica y las redes sociales ampliaron el acceso a información rápida y confiable, consejería, referencias y atención postaborto. Las adaptaciones a la prestación de servicios de atención integral al aborto para mitigar las interrupciones a los servicios durante la pandemia de COVID-19 podrían continuar beneficiando la calidad de la atención, el acceso a los servicios, el monitoreo rutinario, así como la inclusividad y comunicación a largo plazo.

## Background

The COVID-19 global pandemic exposed limitations in comprehensive abortion care (CAC) provision. As defined by the World Health Organization (WHO), CAC includes offering information, induced abortion and management of pregnancy loss/spontaneous abortion, and post-abortion care^1^. Restrictions designed to keep the public safe from COVID-19 infections, such as social distancing, lockdowns, forced clinic closures, and transport restrictions^2^, in many instances, prevented people from accessing sexual and reproductive health (SRH) services, including CAC^3–9^. The potential consequences of this for low- and middle-income countries were noted early in the pandemic^10–13^. In April 2020, the Guttmacher Institute estimated that country-wide lockdowns and disruptions in CAC provision would lead to as many as 3.3 million unsafe abortions worldwide^10,11^. To mitigate, reproductive health advocates pushed for the reclassification of CAC as essential and time-sensitive, and for the expansion of safe channels for the administration of medical abortion (MA) in both clinical and non-clinical settings^14–16^. The WHO’s guidance on maintaining essential health services in the context of COVID-19 recommended reducing barriers that would delay care, especially for vulnerable populations, the minimisation of facility-based care, and forecast an increase in the demand for MA^17^.

Despite limiting CAC provision, the COVID-19 pandemic also created opportunities to expand services. Guided by past lessons from emergency-settings^18^ and advances in telemedicine^19–25^, both international and national non-profit and non-governmental organisations (NGOs) in the field of SRH adapted their CAC programmes to maintain access to services, while keeping their clients, providers, and communities safe from COVID-19^26^. Furthermore, owing to efforts from SRH advocates, policymakers enacted innovative emergency SRH protocols to ensure safe continuity of CAC access within the COVID-19 restrictions^27^. For individuals for whom the COVID-19 pandemic made healthcare facilities difficult to access, the established benefits of self-managed MA became further evident and elevated the option as essential for CAC^28^.

Analyses of the adaptations to CAC services undertaken during the COVID-19 pandemic thus far have reported on high-income contexts^29,30^ or focused on the impact of COVID-19 on the abortion-seeking experience^31,32^. There is limited documentation of specific adaptations implemented for the continuity of CAC services during the COVID-19 pandemic in low- and middle-income settings. To identify and document strategies undertaken to ensure the continuity of CAC service delivery amid restrictions of movement during the COVID-19 pandemic, we conducted a programme evaluation using a case study design to examine adaptations to services implemented in Bolivia, Mali, Nepal, and the occupied Palestinian territory including east Jerusalem (hereinafter referred to as “occupied Palestinian territory” or “oPt”). We sought to explore the facilitators and barriers to these adaptations to inform and support the development and implementation of strategies to safeguard access in these and other settings. The findings contribute to the growing body of evidence on adaptations to CAC service delivery implemented in the context of the COVID-19 pandemic.

## Methods

Programme evaluation systematically assesses the processes and/or outcomes of a programme toward furthering its development and improvement^33^. The approach is not concerned with impact or generalising findings but rather focusses on understanding a programme within its specific context. Programme evaluation using a case study design is appropriate for achieving the above objective because it is well suited to examining contemporary events within their real-life context^34^. The approach is particularly appropriate when attempting to obtain an in-depth understanding of a multi-faceted and complex phenomenon as it naturally occurs^35^. Case study methodology is suitable when there is limited or no control over the development of a naturally occurring event, such as the COVID-19 pandemic, and where there are clearly definable “cases”, as in this instance, different social, economic, and cultural settings^36^. Cases are not selected to be representative of other instances of the phenomenon occurring in a given context. Rather, they are undertaken to understand the uniqueness of an event by exploring it more closely. The approach allows for the inclusion of multiple sources of information to provide an in-depth description of a specific phenomenon^36^. Our specific questions for this case study are:

1. What strategies were undertaken to ensure continuity of CAC services during the COVID-19 pandemic?
2. What were perceived by providers and other stakeholders as the facilitators and barriers to sustaining these adaptations beyond the context of the pandemic?

### Case study settings selection

A working group of partners consisting of representatives of key organisations working towards increasing global access to CAC (Ipas Global, International Planned Parenthood Federation, Marie Stopes International (MSI), Population Services International (PSI), United Nations Population Fund (UNFPA) and the UNDP-UNFPA-UNICEF-WHO-World Bank Special Programme of Research, Development and Research Training in Human Reproduction (HRP)/World Health Organization (WHO)) determined the scope, selected the settings, and provided overall guidance and verification of findings for this case study. This working group was part of a larger network founded in 2020 in response to the COVID-19 pandemic and that has been working collaboratively ever since then. To select settings for this programme evaluation case study, we used an online interactive tool, the *COVID-19 Safe Abortion Response Map*^37^ housing information on 43 programmes taking place in 19 countries. We aimed to include contexts that represented a balance of COVID-19 mitigation strategies for different aspects of service delivery, a mix of public and private sector health care settings, allowed for one case study from each continent, and demonstrated the potential for sustained adaptations beyond the COVID-19 pandemic. The case study settings chosen were Ipas, La Paz, Bolivia; PSI, Bamako, Mali; MSI Reproductive Choices Nepal, Kathmandu, Nepal; and Palestinian Family Planning and Protection Association (PFPPA), oPt (Table 2). The case study organisations were partners of members of the working group.

### COVID-19 restrictions in case study settings

Like most locations around the globe, during the initial months of the pandemic, the case study settings underwent strict lockdowns characterised by mandatory mask-wearing, social distancing, restricted transport and other movement, closure of many formal workspaces, curfews, banning of large gatherings of people, and limited health care to emergencies and the management of COVID-19 (Table 1). It is within the above-described context that adaptations were developed to ensure continuity of CAC services in these settings.

**Table 1.** COVID-19 pandemic restrictions in case study settings*

	Bolivia	Mali	Nepal	oPt
Dates of national lockdown	30 March–30 May 2020	26 March–9 May 2020	24 March–21 July 2020	7 March–20 July 2020
Health care restrictions and SRH legal, policy guideline changes	SRH services were restricted, prioritising COVID-19 care. Attending only emergencies, after the COVID 19 test has been performed.	Due to ongoing civil unrest and the challenges of the pandemic (e.g. the spread of COVID-19 amongst health care professionals), both of which caused sudden and sporadic closure of clinics, services were often unavailable.	Only open for emergencies	Lockdowns restricted access to SRH services, particularly long-term FP methods in governmental premises. Limited access to medical services in general, particularly during lockdowns. The COVID-19 Response Plan in the oPt created inter-agency task forces and formed new strategic relationships across the SRH and water, hygiene and sanitation (WASH) sectors

*As reported by working group of partners.

Prior to the COVID-19 pandemic, services in all sites were typically in-person. In Bolivia, CAC and information provision was provided face-to-face. In Nepal, CAC services could only be provided legally from a government-registered site by a government-certified provider. In Mali, comprehensive SRH information and care was provided for clients in-person with a health care provider. In oPt, abortion is severely legally restricted; both abortion seekers and providers can be criminalised for termination of pregnancy, unless the procedure is necessary to save a woman’s life. CAC is provided as part of harm reduction, including information and counselling as pre- and post-abortion care, and counselling and treatment of incomplete abortion, primarily through in-person services (Table 2).

**Table 2.** COVID-19 safe abortion response map summaries for case study countries.

Bolivia	
Organization	Ipas Ipas is a non-profit, non-governmental organisation that has been working since 1973 in more than 20 countries on three continents: America, Africa and Asia. In Bolivia, it began its activities in 1998, focusing on strengthening the capacity of women, girls and young people so that they can recognise and exercise their Sexual and Reproductive Rights. In Bolivia, Ipas provides in-person provision of information on termination of pregnancy to a largely indigenous population.
Sector	Non-governmental organisation
Area of response	Accompaniment and information provision
Description of response	A theoretical and practical training process was carried out for the agents on the use of medical abortion (Misoprostol and Mifepristone). The pandemic posed a challenge as they could not provide information in person, so they began to use social media for communication.
**Mali**	
Organization	Population Services International (PSI) PSI’s interventions address needs in family planning; safe abortion, maternal, new-born, and child health (MNCH); HIV and sexually transmitted infections (STIs); water, sanitation, and hygiene (WASH); malaria; and primary healthcare (PHC), among others. Abortion in Mali is restricted and legal only to save the life of the pregnant person and in cases of rape and incest. Both medication abortion and surgical abortion are available for post-abortion care and pregnancy termination. Medication abortion is legal through 13 weeks gestation. PSI has trained public and private sector providers on post-abortion care and manual vacuum aspiration and distributes misoprostol.
Sector	Private-not-for-profit
Area of response	Quality assurance
Description of response	To maintain quality services and prevent COVID-19 transmission, PSI/Mali utilised WhatsApp for supportive supervision for PAC and post-abortion contraception.
**Nepal**	
Organization	MSI Reproductive Choices
Sector	Private-not-for-profit
Area of response	CAC service delivery
Description of response	MSI Nepal worked to ensure passage of “Interim Guidelines on Reproductive, Maternal, Newborn, Child and Adolescent Health” that made is legal to provide medical abortion services outside of a certified health facility. Important new provisions include: medical abortion services allowed at home and all licenced chemists allowed to stock and dispense medical abortion products.
**occupied Palestinian territory**	
Organization	International Planned Parenthood Federation Member Association, Palestinian Family Planning and Protection Association (PFPPA) PFPPA operate a harm reduction approach to abortion care, providing women with counselling and accurate information on the safest methods to avoid an unsafe abortion, and post-abortion care and follow-up. Prior to the pandemic, harm reduction services were provided in-person at PFPPA clinics.
Sector	Public
Area of response	Digital health interventions
Description of response	During the COVID-19 pandemic and associated lockdown restrictions that prevented women from accessing in-person services at clinics, PFPPA established a toll-free call centre which provides consultations, counselling, referrals and follow-up, including consultation for abortion care through a harm reduction approach, ensuring that women are provided with accurate information.

### Interviews with key informants

We conducted semi-structured interviews between October 2021 and January 2022 with key informants from NGOs in each case study setting. We aimed to conduct two interviews with service providers, and one interview each with facility managers and community access coordinators in each setting to obtain a range of perspectives depending on organisational role. Specific informants were selected by the in-country programme director for each organisation. The interviews explored changes to service delivery, client characteristics, facility management, provider supervision, service quality during the pandemic, and potential for scaling up or sustaining any adaptations in the future. The second author (SC) carried out the interviews and the third author (LF) transcribed the recordings. We organised one group of in-depth interviews with two providers in oPt due to logistical and language constraints. The remaining interview was an individual discussion with one facility manager. Each interview was approximately 45–60 minutes long and simultaneous translation was provided in Spanish in Bolivia and in Arabic in oPt. The interviews from Bolivia, Nepal, and oPt were subsequently transcribed into English and those from Mali transcribed into French for analysis, given the second author’s French language skills.

### Ethics

On behalf of the working group of partners, the WHO and PSI submitted the programme evaluation to their respective Ethical Review Boards (Research Project Review Panel (RP2) and PSI Research Ethics Board) in August 2021. The Boards determined that, as participants were contributing on behalf of their organisations, and this programme evaluation case study design did not constitute human subjects research, no Institutional Review Board (IRB) approval was required. Prior to interview, each informant was briefed on the objective of the evaluation and given the opportunity to ask clarifying questions. Each informant provided oral consent to be interviewed and have the discussion recorded. Informants were also made aware that all data would be anonymised, participation was voluntary, and there was no direct benefit to individuals for their participation. Finally, all informants were told that they could end the interview at any time without repercussion.

## Data analysis

The primary author, a trained qualitative researcher, analysed the data from key informant interviews using a cross-case, data-driven, inductive thematic analysis approach and with contributions from the working group of partners who are experts in the delivery of CAC programmes^34–36^. The primary author translated transcripts to English where necessary using DeepL software (https://www.deepl.com/translator) and then read all the transcripts to allow for immersion and familiarity. Initially, the primary author developed an *a priori* and inductive coding framework based on line-by-line coding and discussed this with the working group of partners before it was applied to the transcripts. The coding framework was refined using constant comparison to develop conceptual themes. We searched across data from each setting and informant category for commonalities, differences, and patterns in adaptations of CAC service delivery in the context of COVID-19 to draw out key lessons and strategies and organised these in Microsoft Excel (https://www.microsoft.com/en-us/microsoft-365/excel). The working group of partners met regularly to discuss emerging themes and contributed to the final analysis. Finally, the findings were shared with participating key informants for confirmation and validation. No changes were made to the findings as a result of the confirmation and validation process.

### Findings

We conducted virtual individual or group interviews with 16 key informants in Bolivia (*n* = 4), Mali (*n* = 4), Nepal (*n* = 4), and oPt (*n* = 2) (Table 3). In our analysis of key informant interviews, we identified four overarching themes related to adaptations to CAC service delivery, as defined by the WHO^1^: (1) the emergence of the use of digital technologies, (2) efforts toward home and community outreach, (3) health worker optimisation, and (4) further consideration of groups in vulnerable situations. Each broad theme was examined in depth and interpreted across settings. We discuss these in turn below.

**Table 3.** In-depth interviews with key informants.

Key informant type	Nepal (*n*)	Mali (*n*)	oPt (*n*)	Bolivia (*n*)	Total
Facility manager/Access coordinator	1	1	1	1	4
Provider	2	2	2	2	8
Representative from a community or NGO that supported the adaptation	1	1	1	1	4
Total	4	4	4	4	16

### The emergence of the use of digital technologies

In many sectors, social distancing and other restrictions during the pandemic required that previously in-person interactions, pre-COVID-19, become virtual using digital technologies, such as teleconferencing, social media, and online messaging platforms. This was also true for the delivery of CAC in the case study settings.

In oPt, PFPPA providers adopted digital approaches to conduct counselling for contraception, abortion care under the harm reduction approach, and post-abortion care. PFPPA providers established a hotline and used social media to maintain SRH services on-demand, carried out interactive information-sharing sessions among groups, and referrals of clients to trained providers and collaborating physicians. To maintain confidentiality, in the group sessions PFPPA providers encouraged attendees to message them privately. In cases of requests for CAC services, PFPPA providers would give harm reduction counselling and consultation services via digital communication channels, which included information on the safest methods of abortion available in their context and advice on signs of complications and post-abortion care.

*“We care a lot about privacy and confidentiality. […] if anyone wants to have a private consultation or counselling, they can talk to me on [a messaging application that uses the internet to send encrypted messages] or they can even send a private message via [social media].”* (Provider, PFPPA, oPt)

In Bolivia, lockdowns and restrictions on travel instituted during the early days of the pandemic and the shifting of focus in the health sector towards treating COVID-19 patients meant that abortion providers were required to move their interactions with clients online. Ipas Bolivia providers worked with community-based coordinators to set appointments using a messaging application that uses the internet to send encrypted messages, via telephone or teleconference.

*“For example, in terms of [a messaging application that uses the internet to send encrypted messages], we were very hesitant at first because we were not sure what kind of person we were getting, who was going to be coming but through a series of interview questions we were able to sort of better get to that and be able to get them an appointment.”* (Provider, Ipas Bolivia, Bolivia)

To continue to engage with the community, information about Ipas Bolivia services was posted on social network platforms. Respondents perceived an increase in engagement with potential clients using social media during this period. They attributed the continuation of MA information provision during the pandemic to the “safety”, “privacy” and “comfort” afforded by social media platforms.

*“I think more than anything that the use of social networks helped to make women feel comfortable coming forward. Because when you are talking face-to-face it is one thing, but when you are using your phone you feel safer to talk about private things … ”* (Community Access Coordinator, Ipas Bolivia, Bolivia)

Like other settings in this case study, once connected with clients, Ipas Bolivia providers continued to use digital platforms to arrange for clinic visits, prescribe medication or to give advice, even to those outside of their catchment area.

In some settings, restrictions on movement required providers to explore new mechanisms for supervision and reporting. Rather than the usual in-person reporting, providers submitted their monthly service statistics by a messaging application that uses the internet to send encrypted messages, or by email for supervision and for inclusion in HMIS systems. This was done by either taking a photograph of the medical record manually or by entering the medical record data into the phone or computer by hand.

*“ … so we had to also adapt to this [virtual supervision], because before the supervisor would go to the community and she would pick up all the registries or documents with that information. But now, we could not do that, so we would use Apps … and the persons in the community would take a photo of this registry and send it as a PDF.”* (Community Access Coordinator, Bolivia)

In oPt, using digital technologies for counselling and reporting was described as “easier” than the in-person procedures used prior to the pandemic. Communities are already using digital technologies for personal purposes and therefore can also use social media to seek and offer services faster and with less effort.

*“ … after COVID-19 and after this reliance on technology, it made services easier. For example, if she needs papers from the court, before we used to ask her to come in and deliver it by hand. But now, she can send it by social media platform.”* (Women’s Center for Legal Aid and Counselling, oPt)

### Efforts toward home and community outreach

As during COVID-19 in-facility care was no longer a possibility, some providers elected to either connect with other providers located in communities or travel there themselves to ensure the continuation of services. In Nepal, MS Ladies (providers at MSI Reproductive Choices) added home visits to the facility-based services.

*“ … MS Ladies provided MA to clients in their home. They created a very supportive environment to access safe abortion services and the other part is that they [work] side-by-side continue to provide abortion services in [the] facility … ”* (Community Associate Representative, MSI Nepal, Nepal)

Providers working in facilities made use of existing networks within communities to enable the sharing of information, setting of appointments, and delivery of services. In Bolivia, local Ipas coordinators booked appointments for women; in Mali, trained members of the community served as “interlocutors” between the communities and the MSI service provider; in Nepal, government senior health volunteers and youth mobilisers in the community made referrals for services at PSI and to ensure individuals were aware of the MA services available at local pharmacies; and in oPt, PFPPA providers brought contraceptives to clients’ homes.

*“Because unsafe abortion is also when there is no one to confide in. But if you have someone in the community who is known […], even if it's at night on the phone, someone will call them anyway to explain their situation, and I think they can be a good support.”* (Youth Association, Mali)

Home and community outreach efforts leveraged and strengthened existing community networks to ensure a continued flow of information and services from facilities and city centres to communities during lockdown.

### Health worker role optimisation

In Nepal, prior to the pandemic, surgical and MA services were widely available, but only through registered public, private, and NGO facilities. However, in May 2020 a new legal provision, previously championed by stakeholders working closely with the government of Nepal, unprecedentedly allowed for trained health service providers from NGOs and private organisations to provide MA services in pharmacies and at clients’ homes. MSI Reproductive Choices providers worked with pharmacists to provide on-site counselling and MA provision to clients presenting at the pharmacy.

MSI Reproductive Choices providers used the private pharmacy examination rooms to assess women’s eligibility for MA services and counsel them on the process, side-effects, signs of complications and where to seek follow-up care if needed. If a woman was eligible for MA, MSI Reproductive Choices providers would dispense misoprostol or the combination regimen of mifepristone and misoprostol at the pharmacy and follow up with the women at home via telephone.

The respondents noted that, following the change in the law, a greater number of women were requesting MA services at pharmacies compared to the pre-pandemic period.

*“With the pharmacies, previously we used to have 2–3 clients in a month, but we have been receiving more clients since the partnership was good as we went there on their request.”* (Provider, MSI Reproductive Choices, Nepal)

In addition to collaboration with pharmacists, Auxiliary Nurse Midwives (ANMs) working in MSI Reproductive Choices facilities in Nepal would also travel to communities to provide services.

*“After the screening, ANMs would pack the MA commodities in a backpack and would go into the community to provide services.”* (Facility manager, MSI Reproductive Choices, Nepal)

In Bolivia, lay health care workers were trained to provide information, counselling, and referrals in the community during the COVID-19 pandemic.

*“ … these are community agents, that may attend university or may belong to organizations, they are not necessarily medical students, but they are trained to provide information on medical abortion and other methods for family planning.”* (Community Access Coordinator, Ipas Bolivia, Bolivia)

In oPt, PFPPA social workers are trained to provide abortion harm reduction counselling and consultation. Although this was a strategy used pre-pandemic, during COVID-19 social workers played a central role in providing CAC.

Sharing the delivery of CAC services with a wider range of trained health care professionals in these contexts was an adaptation that not only allowed for the continuation of services, but also potentially broadened it.

### Considerations for groups in vulnerable situations

The constraints, pressures, and concerns related to the COVID-19 pandemic further marginalised and highlighted the health inequities of groups already in vulnerable situations^38^. The key informants in all settings in this case study described increases in gender-based violence (GBV), particularly among groups such as the LGBTQ+ community, increased support, and health care needs among individuals living in rural communities, and the novel steps they took within their programmes to meet the needs of these vulnerable populations.

In oPt, PFPPA integrates GBV screening into all their counselling services to identify victims and provide tailored support and care according to their needs.

*“There are statistics which show a huge increase of GBV cases during COVID-19. […] We did a kind of comparison between the year 2020 and other years with regards to GBV and found it had increased”* (Member, Community Association (Legal advice), PFPPA, oPt)

In Bolivia, Ipas created a social media campaign and MA referral system that focused on educating people about their legal right to MA in cases of sexual violence. Persons who were interested in getting additional assistance were confidentially linked with providers through private messenger or a messaging application that uses the internet to send encrypted messages and phone calls.

*“We trained our agents on medical abortion and we trained them about sexual violence as well … . There is a constitutional law here, 0206, and you know it protects victims of violence and rape. Under those circumstances, we are able to then connect them so they could seek services to allow them to end their pregnancies.”* (Community Access Coordinator, Ipas Bolivia, Bolivia)

The adaptations to service delivery were viewed as increasing access to communities that had been traditionally underserved and, in the context of the COVID-19 pandemic, even further difficult to reach.

*“Through these adaptations everyone was able to reach people who could not get the service before from marginalized and far away and poor societies.”* (Community Association, PFPPA, oPt)

### Facilitators and barriers to continuing adaptations in the future

In general, the informants were positive about the adaptations made to their programmes during the COVID-19 pandemic and were hopeful that the measures would continue once the crisis had passed. For example, in Bolivia the Community Access Coordinator was enthusiastic about continuing to use digital technologies:

*“[…] we are also going to continue with the social media with the networking, because some people actually feel more safe, for example they can just call and they don’t need to see each other. People feel sometimes safer that way too sometimes..”* (Community Access Coordinator, Ipas Bolivia, Bolivia)

However, they recognised that this approach may not be suitable in smaller, less populated municipalities where access to the internet is limited. In those locations, they would have to return to offering in-person services.

In Mali, the PSI Mali informant recognised how digital technologies increased equality in service access and supported continuation:

*“Yes, why not because my wish is that everyone can have access to these kinds of services from where they are. So if from your village, from your house, from where you are, from your workplace, you can have access to certain types of information, without having to move, this will allow clients to be at the center of their health. I think that if this continues it will be a good thing.”* (Youth Association, PSI Mali, Mali)

However, respondents also noted that some providers have limited skills to use digital technologies and will need additional training and support to continue the approach. Furthermore, there were costs related to internet connectivity which many providers and facilities could not afford, and some settings, for example in rural areas, did not have the infrastructure to handle a large amount of internet traffic. Digital technologies also potentially increase challenges of confidentiality and online protections, which are not currently adequately addressed in health systems.

*“ … so more difficult also because there are some providers who do not even master the phone let alone the machines. […] The computers I mean.”* (Provider, PSI Mali, Mali)

While the pharmacy adaptation in Nepal appeared to increase crucial MA access during the pandemic, the informants noted that there is a need to conduct mass MA training for pharmacists and develop quality assurance and monitoring mechanisms before the task-sharing approach can be adopted as a permanent model of care and scaled-up in the post-pandemic setting.

*“I am in favor that it [pharmacy adaption] should be available but only with trained pharmacists (in MA) and not in the current way.”* (Provider, MSI Reproductive Choices, Nepal)

Although many pharmacies are already informally offering CAC, the legal barriers to formal pharmacy provision must be addressed.

Finally, some informants described needing additional support, resources, and the “necessary tools” to continue with the adaptations. This was in reference to materials, such as brochures and posters, but also smartphones and other electronics required for online communication.

*“But it is difficult to have the necessary tools, for example I only have one phone and I think this one will only last for two months at best. Maybe one month. But we don’t, I don’t have the tools, I don’t have a computer. If you don’t have the tools you can’t do your job, its very important to have the right tools.”* (Community Activist, Ipas Bolivia, Bolivia)

However, the digital technologies used to continue CAC during the COVID-19 pandemic relied on tools, such as smartphones, that much of the population is already familiar with and has in their possession.

Despite the need for additional resources and training to continue the adaptations, the key informants unanimously agreed that the steps taken to maintain access to safe abortion during the pandemic should continue beyond COVID-19, as they have the potential to increase access to services.

## Discussion

This programme evaluation illustrates how, despite the challenges COVID-19 presented, providers in Bolivia, Mali, Nepal, and oPt were able to adapt their programmes to maintain services. This dexterity in time of crisis was characteristic of many health care providers during the pandemic^39^. The adaptations presented above were perceived by key informants as beneficial to populations facing difficulties during the COVID-19 pandemic and possibly useful for increasing quality and accessibility in future service provision. Although the adaptations described pertained to CAC, including surgical methods, in each case study setting, there was a primary focus on the use of MA. As has been noted elsewhere, MA lends itself to demedicalisation and the pandemic appears to have provided an opportunity to expand access^31^.

Globally, the use of digital technologies to inform clients of services, provide counselling and consultation, offer support and supervision to providers, and transmit routine data has been critical to maintaining safe and effective access to MA and other health services throughout the pandemic^19–25^. Digital approaches supported clients’ access to contraception and CAC during the pandemic, and enabled providers to reach already underserved and marginalised communities. Where there is lack of access to services and high trust of digital technologies, even in remote areas and among the underserved, there is great potential for enabling CAC service seekers to shape the type of support received.

Even though benefits were recognised in terms of data quality and timeliness, some providers’ unfamiliarity with digital tools and email, and lack of access to smartphones and other electronics hindered the current and long-term use of digital technologies. Furthermore, there may be important implications for confidentiality and data protection with the increased use of digital technologies^40,41^. Although the messaging applications used in most of the sites were encrypted to ensure privacy, social media and other digital technologies are not designed for data security and could pose important risks. The growth of digital technologies, such as telemedicine, and other approaches to self-care are critical for meeting the demand for services in times of crisis where health care utilisation is challenged, as in the case of the COVID-19 pandemic^42^. In fact, some argue that the provision of health care remotely where possible is the duty of health care systems to reduce equities in access^43^ Unequal access, data protection, and unintended consequences are ongoing challenges to the inclusion of digital technologies in health care that must be addressed^40,41,44–46^. But, this does not minimise the effectiveness of pragmatic solutions such as these in times of need. These adaptations can serve as prototypes to be tested, piloted, and improved for future scale-up of safe and secure digital interventions in these settings and elsewhere.

Providers were able to leverage pre-existing trust, relationships, and networks among the communities they served to bring MA services into the community and expand their outreach through strategies including home visits. For example, in Bolivia, community outreach workers and campaigners sought to build trust, making sure MA services were inclusive and available to all. In Mali and Nepal, existing networks in the community facilitated outreach and ongoing communication for the delivery of safe abortion services during lockdowns and thereafter.

The importance of legal and institutional support to guarantee safe and appropriate care for all people seeking a CAC has been widely recognised globally^47^. In Nepal, access to MA has been increased by new policies, practices, and the enactment of new laws, as well as task-sharing with mid-level health care providers, including pharmacists^48^. While there is a need to expand pharmacists’ technical knowledge of MA services and products, this adaptation demonstrates that there is client demand and provider opportunity for MA services to be safely delivered through this channel and for women to be provided with appropriate information to self-manage if they so choose.

The COVID-19 pandemic was associated with a documented increase in GBV^49^. Although interviewees were not able to make a direct link with the demand for abortion due to a lack of data, global evidence suggests that a rise in GBV during the pandemic may lead to an associated rise in demand for CAC services. As a result of an observed increase in GBV in their communities, providers were able to adapt strategies and models of care to meet the increased needs of people vulnerable to GBV, including ensuring access to CAC. This aligns with growing evidence from the UK suggesting that telemedicine for early MA may also increase opportunities for safeguarding^50,51^.

NGOs and other organisations providing MA services frequently emphasise the inclusivity of their reach, for example, to those in humanitarian settings or to adolescents^18,52^. The adaptations highlight an opportunity for organisations to strengthen their focus, reach, and communication to engage with people in vulnerable settings and draw upon grassroots’ insights and strategies, such as partnership strengthening.

### Strengths and limitations

This case study programme evaluation offers in-depth information about how organisations adapted service delivery within the constraints of the global pandemic to ensure continued CAC. The data collected represents the perspectives of providers in diverse low- and middle-income contexts. These findings illustrate how adapting existing tools innovatively can broaden access to CAC in the modern context.

However, as the purpose of this programme evaluation case study was to document strategies undertaken to ensure the continuity of CAC delivery during the COVID-19 pandemic, it cannot speak to the effectiveness of the adaptations. Furthermore, people seeking CAC themselves were not included as key informants in this case study, therefore, we cannot know whether they found the services acceptable and of quality. Lastly, as noted above, the informants were chosen by the country office directors. There are therefore likely to be inherent biases in their responses which may seek to overly focus upon positive aspects of programme adaptations. It should be noted that some country programmes implemented further changes to aspects of service delivery in addition to the ones described here. In the constantly evolving environment of the COVID-19 pandemic, ad hoc adjustments to services were regularly made in response to concerns perceived by providers and other stakeholders. What is reported here is a snapshot of what occurred during that period of flux. Finally, adaptations to services outside the formal health system were difficult to capture due to risks and legal sensitivities, for example within feminist networks, but we focus on the adaptations detailed above to highlight the continued resourceful and innovative provision of CAC during the pandemic.

## Conclusion

COVID-19 presented a considerable number of challenges for the continued delivery of CAC services. Further research would benefit from ascertaining client perspectives from all communities who may wish to use MA services, and how these can be accommodated in adaptations both during the pandemic and beyond. Going forward, the impact of these adaptations on CAC service delivery beyond the pandemic should also be examined. However, it is clear that COVID-19, although extremely challenging for CAC provision, also catalysed opportunities for sustained and improved MA service delivery which may benefit service quality of care, access to care, and routine monitoring, as well as inclusivity and communication in the longer term.

## Data Availability

The datasets used and/or analysed during the current study are available from the corresponding author on reasonable request.
